# Two-dimensional Czochralski method for large-scale single-crystal MoS_2_

**DOI:** 10.1093/nsr/nwaf127

**Published:** 2025-04-08

**Authors:** Kunpeng Si, Yongji Gong

**Affiliations:** Tianmushan Laboratory, Beihang University, China; School of Integrated Circuit Science and Engineering, Beihang University, China; Tianmushan Laboratory, Beihang University, China; School of Materials Science and Engineering, Beihang University, China

Two-dimensional (2D) materials, such as MoS_2_ and hexagonal boron nitride (hBN), are poised to redefine the future of electronics by enabling ultra-scaled transistors, flexible devices, and energy-efficient integrated circuits. Their atomic thickness and exceptional electronic properties position them as key enablers for extending Moore's Law beyond the limits of traditional silicon-based technologies [1]. Nevertheless, their industrial-scale implementation remains constrained by limitations in the batch production of large-scale high-quality 2D materials [2]. Thus, the capacity to synthesize large-scale 2D materials represents an essential prerequisite for the fabrication of next-generation integrated circuits. Moreover, the presence of grain boundaries in 2D materials induces carrier scattering, significantly degrading the performance of electronic devices [3]. Hence, stringent control of grain boundaries and the production of superior monocrystalline 2D films are imperative.

Contemporary research has demonstrated the successful synthesis of wafer-scale MoS_2_ single crystals, which is merged from micrometer-sized domains aligned in a single orientation using evolutionary chemical vapor deposition (CVD) [4]. However, the multi-seed growth method is often limited by imperfect domain splicing, leading to translational grain boundaries. The single nucleus growth method, which has made progress on graphene, addresses this issue at a mechanistic level [5]. However, due to the difficulty of suppressing nucleation density, large-area single-layer transition metal dichalcogenides (TMDCs) monocrystals could not be achieved through analogous methodologies [6,7]. He *et al*. introduced an innovative 2D Czochralski (2DCZ) methodology [8], which entails establishing an extensive 2D liquid precursor on a wetted substrate, thereby diminishing nucleation density and facilitating the growth of centimeter-scale single-domain MoS_2_. The resulting single-domain MoS_2_ crystals can reach up to 1.5 cm, exhibiting an exceptionally low defect density of 2.9 × 10^12^ cm^−2^, rendering them highly suitable for high-performance field-effect transistors (FETs).

**Figure 1.**
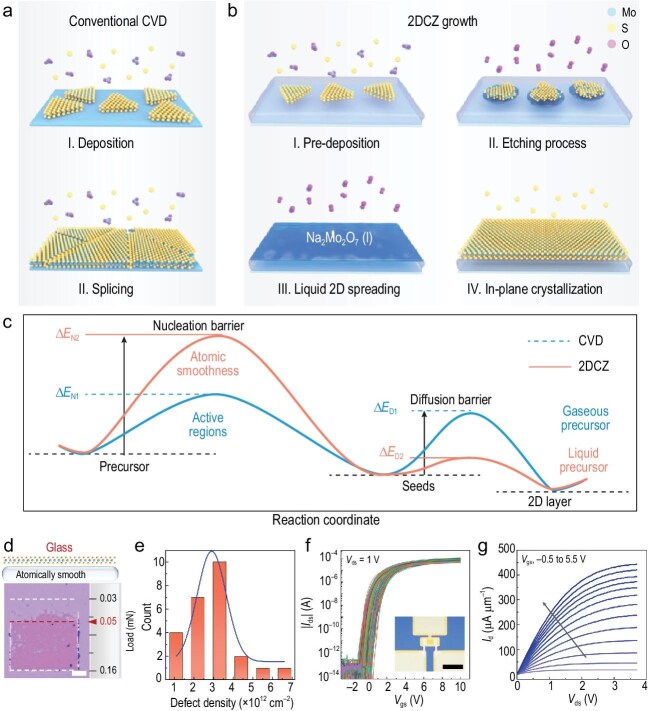
(a) Schematic of conventional CVD process for MoS_2_ growth. (b) Schematic overview of the 2DCZ growth process. (c) Comparison of the progress of energy fluctuation between CVD growth and 2DCZ. (d) Results of the nano-scratch test on glass; mapping areas are 40 × 40 μm^2^, and the top shows a schematic of the surface. Scale bars, 10 μm. (e) Statistical bar graph showing the defect density observed in the HAADF-STEM images. (f) Transfer characteristics of 185 FETs (out of 192); the inset shows an optical micrograph of a single FET. (g) Output characteristic of the short-channel FET, with V_gs_ from −0.5 to 5.5 V with a 0.5 V step. Reprinted with permission from Ref. [8]. Copyright 2025, Springer Nature.

The researchers accomplished large-scale 2D single-domain growth by simultaneously reducing the nucleation density (increasing the nucleation barrier) and accelerating growth rates (reducing the diffusion barrier). While traditional CVD methods rely on adsorption, random nucleation, and splicing at the vapour-solid interface (Fig. 1a), the 2DCZ method prefabricates a 2D liquid precursor film and employs a chemical process to facilitate the liquid-solid transformation (Fig. 1b). First, the atomically smooth and defect-free surface of the molten glass significantly raises the nucleation barrier, thereby reducing the density of nucleation sites (Fig. 1c). Subsequently, the 2D precursor liquid film is then formed through a two-step reaction. The Mo precursor undergoes pre-deposition and etching via precise control of O_2_ and S vapor pressures. A eutectic reaction and liquid-liquid phase separation on the molten glass substrate then produces a stable 2D liquid precursor film. The interface between the 2D liquid film and molten glass manifests a high surface tension, which promotes the diffusion of the liquid precursor and forms distinct liquid-liquid interfaces. Ultimately, the 2DCZ method eliminates the adsorption process, with S vapour triggering the final liquid crystallization. The pre-diffused precursor exhibits a significantly lower diffusion barrier on the substrate compared to CVD methods, enabling the rapid growth of MoS_2_ crystal domains. As a result, the combination of ultra-low nucleation density (2.9 × 10^12^ cm^−2^) and ultra-fast growth rate (75 μm s^−1^) effectively eliminates large-scale grain boundaries.

The high crystallinity and potential for application of MoS_2_ samples were validated through several approaches. MoS_2_ films produced by the 2DCZ method could be gently exfoliated in deionized water, benefiting from the smooth surface of the glass substrate and weak coupling between the sample and the glass, which manifested in substantially reduced adhesion force compared to conventional substrates (Fig. 1d). High-angle annular dark-field scanning transmission electron microscopy (HAADF-STEM) imaging at atomic resolution quantified sulfur vacancy densities across multiple regions. Figure 1e illustrates the statistical histogram of sulfur vacancy defect densities across 25 different regions, revealing a remarkably low variance density of 2.9 × 10^12^ cm^−2^. These superior qualitative attributes facilitated an impressive 96.4% yield (185/192) in the fabrication of centimeter-scale FET arrays, exhibiting exceptional performance characteristics (Fig. 1f). The average mobility of the FET arrays was 55 cm^2^ V^−1^ s^−1^, with remarkable consistency in device functionality. Device uniformity was exceptional, with variations in mobility, subthreshold swing (SS), and threshold voltage (V_th_) limited to 15.9%, 16.8%, and 15.3%, respectively. For short-channel (480 nm) FET devices, a saturation current density of 443.8 μA μm^−1^ was achieved (Fig. 1g), with the best mobility of a single FET reaching 105.4 cm^2^ V^−1^ s^−1^. Furthermore, an integrated logic gate array was designed based on pseudo-NMOS logic and successfully realized. This demonstrates the applicability of the high-quality, highly uniform MoS_2_ in integrated circuits and significantly advances the application of 2D devices.

In summary, this innovative study provides a novel synthesis method for large-scale single-crystal 2D materials. The research demonstrates that by constructing a wetted substrate with a 2D liquid precursor, the Czochralski method can be adapted for 2D materials, ultimately enabling the growth of 1.5 cm monocrystalline MoS_2_ domains. This pioneering fabrication method overcomes the challenge of translational grain boundaries and could revolutionize the industrial-scale production of 2D materials. By enabling centimeter-scale single-crystal growth with high reproducibility, the 2DCZ method offers a scalable pathway compatible with existing semiconductor manufacturing workflows, potentially reducing production costs and accelerating the integration of 2D materials into next-generation integrated circuits. Furthermore, the implementation of *in situ* detection techniques has enabled profound insights into the reaction dynamics of 2D liquid precursors, substantially advancing our understanding of 2D material growth mechanisms.
